# Microalgae, Cell Factories for Antimicrobial Peptides: A Promising Response to Antibiotic Resistance

**DOI:** 10.3390/antibiotics14100959

**Published:** 2025-09-24

**Authors:** Malika Mekhalfi, Sabine Berteina-Raboin

**Affiliations:** Institut de Chimie Organique et Analytique (ICOA), Université d’Orléans, UMR-CNRS 7311, BP 6759, Rue de Chartres, CEDEX 2, 45067 Orleans, France

**Keywords:** microalgae, cell factories, antimicrobial peptides, antibiotic synthesis, natural sources, resistance, bioproduction

## Abstract

The prevalence of infectious diseases is steadily increasing. If left untreated, they can lead to more serious health problems. Antibiotics currently available on the market are facing growing resistance, prompting the development of increasingly powerful antibacterial molecules. One alternative currently under investigation is the use of antibacterial peptides, whose mechanisms of action differ from those of conventional drugs. These peptides are produced naturally by all living organisms and can also be synthesized. However, as peptide chains become longer, synthesis and purification become increasingly complex and laborious. For decades, antimicrobial peptides have been synthesized on polymer supports using automated systems. Unfortunately, longer chains tend to fold more, preventing access of reagents within the cross-linked polymer network. Recombinant production of antimicrobial peptides has been achieved in various organisms called “cell factories,” allowing for more sustainable synthesis. Recently, microalgae have emerged as a promising and sustainable alternative for the production of antimicrobial peptides. They are inexpensive, easy to cultivate, and capable of producing biologically valuable molecules, offering a potential solution to antibiotic resistance. This work reviews the current state of these “cell factories” and examines the advantages and limitations of microalgae for the future of biopharmaceutical production.

## 1. Introduction

Although the incidence of infectious diseases has declined due to the constant development of new broad-spectrum and targeted antibiotics, they remain a major public health problem. However, antibiotics misuse in terms of treatment duration [1], as well as their intensive use, have generated numerous resistances which are becoming a real problem in the management of certain infections [2,3,4,5,6,7]. Bacteria are highly adapted to their environment, enabling them to develop intrinsic resistance defense mechanisms against antibiotics [5,6]. The World Health Organization (WHO) has published data attesting to the major impact of antibiotic resistance on public health, estimating the associated mortality at 5 million deaths worldwide [8]. According to the WHO, this mortality rate is projected to rise substantially by 2050, potentially doubling. A great deal of research is therefore devoted to limiting bacterial transmission and finding ways of preventing it. Various routes have been explored, such as better management of medical prescriptions, research to limit the development of resistance genes, and natural access to new antibiotics. With regard to the latter area of interest, we have already identified natural antibiotic molecules present in a balanced diet that generate much less resistance while preserving the gut microbiota. Another rapidly expanding possibility is the use of monoclonal antibodies, which have various modes of action. As G. Desoubeaux [9] points out, health authorities have already approved seven of these antibodies for the treatment of viral infections, post-antibiotic colitis caused by *Clostridium difficile* [10] and atypical hemolytic uremic syndrome caused by enterohaemorrhagic *Escherichia coli* [11].

Still in the field of natural resources, we will now turn our attention to a promising and innovative alliance between the bioproduction of biomedicines and microalgae. These three complementary forces could become a key strategy in the fight against a very real threat: antibiotic resistance.

## 2. Results and Discussion

**Figure 1:**
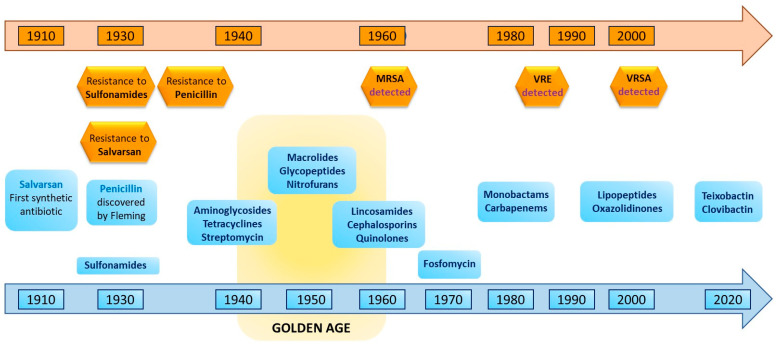
Timeline of the discovery of antibiotics and emergence of these multidrug resistances.

This section provides a brief overview of the history of antibiotics. It begins with molecules originally produced naturally by various organisms such as bacteria and fungi. Alexander Fleming was awarded the Nobel Prize in 1945 for his discovery of penicillin isolated from *Penicillium notatum* in the early 20th century (1928). Rapid progress then led to the discovery of entire families of molecules, such as tetracyclines, marking the “boom” of antibiotics. Each of these discoveries pushed the boundaries of what we could treat. However, as Figure 1 shows the rate of new discoveries slowed significantly from the 1980s and 1990s onwards.

Very few new classes of antibiotics have been developed, while at the same time bacteria have begun to resist the effects of these molecules. As mentioned above, one of the main reasons for this is human use of antibiotics. They are often prescribed too easily and in excessive quantities, and self-medication is common outside the medical field [12,13,14]. In fact, they have become a kind of infrastructure on which our agri-food system relies, particularly in intensive livestock farming. Faced with this negligence, bacteria have rapidly developed a diverse set of genetic mechanism to fight antibiotics through various mechanisms included point mutations. Then, as they reproduced, these bacteria exchanged genetic material [15,16,17], a process called horizontal gene transfer, which allows them to constantly develop new forms of resistance. This evolutionary creativity makes the fight against antibiotic resistance particularly complex. Since the 1990s, there have been few innovations in this field, with the exception of a recent discovery in 2023 following work by researchers from various German and American universities who succeeded in isolating clovibactin from a “dark matter bacterium” that could not previously be cultured. This molecule is capable of fighting multidrug-resistant bacteria through to the combination of three simultaneous targets. It was preceded in 2020 by the discovery of teixobactin, which is active against Gram-positive pathogenic bacteria and was considered a major breakthrough in the search for new antibiotics. Clovibactin targets pyrophosphate anchored in the lipids of the bacterial membrane at several precursors involved in peptidoglycan synthesis, thereby blocking the synthesis of this cell wall. Its sequestration action on precursors allows it to bind with high efficiency and selectivity, contributing to the fact that no resistance has yet been developed. Clovibactin causes greater lysis than teixobactin, which had already been demonstrated, but the rest of their actions are fairly similar. Markus Weingarth et al. [18] showed that clovibactin was effective against methicillin-resistant *S. aureus* (MRSA) strains, as well as against *E. faecalis* and *E. faecium*, which are vancomycin-resistant *enterococci* (VRE), and many others in vitro and in vivo.

**Figure 2:**
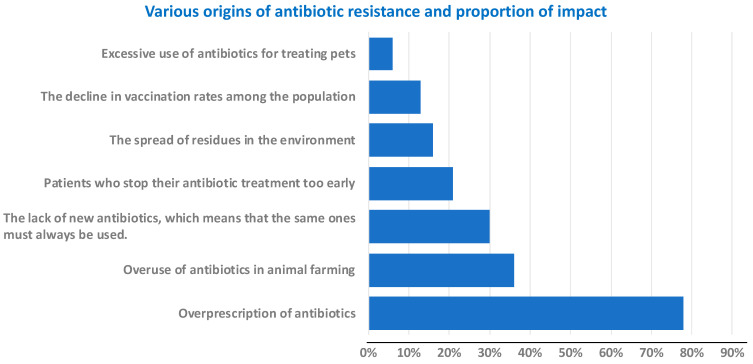
Various origins of antibiotic resistance and proportion of impact [19].

In addition to the recent discovery of these two new antibiotics, the various causes of resistance to other antibiotics and their impact are summarized in Figure 2 [19].

The consequences for human health are clear: certain bacteria have become resistant to several antibiotics, or even to all those available on the market. This is the case with MRSA (methicillin-resistant *Staphylococcus aureus*) and VRE (vancomycin-resistant *Enterococcus*), and more recently VRSA (vancomycin-resistant *Staphylococcus aureus*), collectively referred to multidrug-resistant bacteria. Today, they are found not only in private healthcare but also in hospitals, which is more problematic because they cause infections that are very difficult to treat. See the timeline of the emergence of these multidrug resistances in Figure 1 (orange timeline).

**Figure 3:**
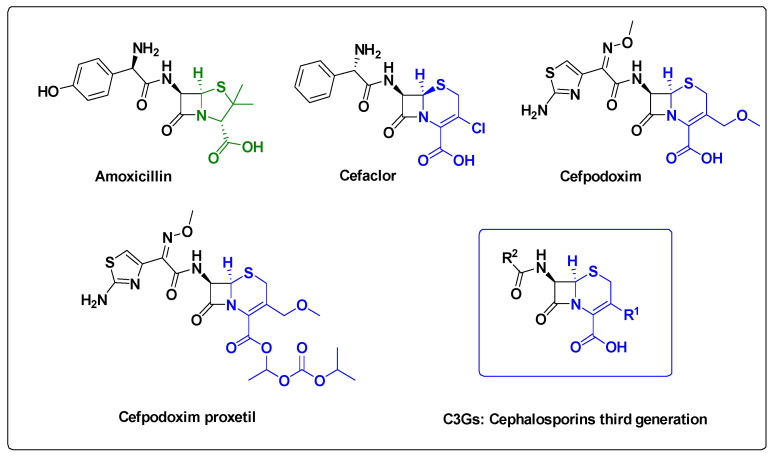
Common β-Lactams, penicillins and cephalosporins.

Unfortunately, it is not just a question of new strains appearing, even within the same bacterium, resistance mechanisms can evolve. Take the concrete example of *Escherichia coli*, a very common bacterium that causes urinary tract infections, among other things. For several years now, there has been a gradual and alarming increase in its resistance to various antibiotics. It should be noted that resistance to penicillin and other antibiotics in the same β-lactam family may also be due to the evolution and diversity of β-lactamases. These enzymes hydrolyze these antibiotic families, such as cephalosporins, monobactams and carbapenems [20]. These molecules are quite constrained and highly sensitive to hydrolysis. Various mechanisms of antibiotic resistance have been examined in detail by Munita and Arias [15]. Commonly prescribed cephalosporins, although their spectrum is fairly narrow, have evolved from amoxicillin^®^ (first generation) to cefaclor (second generation) then to cefpodoxime (third generation) and its precursor ester, cefpodoxime proxetil (Figure 3), which are semi-synthetic compounds.

For example, third-generation cephalosporins (C3Gs, Figure 3), antibiotics commonly used to treat urinary and abdominal infections, are becoming less effective against *E. coli.*

**Figure 4:**
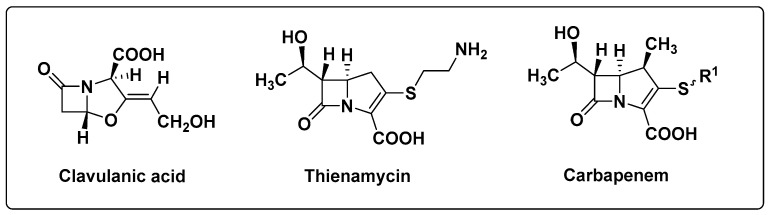
Clavulinic acid, Thienamycin and Carbapenem general structure.

Clavulanic acid [21], thienamycin [22] and carbapenems [23] were developed to fight the emergence of β-lactamase-resistant bacteria (Figure 4). These are β-lactamase inhibitors, which are broad-spectrum antibiotics.

Resistance is not static: it evolves, takes hold, and progresses, meaning that treatments that worked very well 10 or 15 years ago are now sometimes completely ineffective. Antibiotic resistance knows no borders: it spreads everywhere. According to the WHO, in 2024, 24 multidrug-resistant pathogens were responsible for approximately 1.27 million deaths worldwide in 2019. This mortality is higher than that resulting from HIV or malaria. In Europe, an estimated 30,000 deaths each year are linked to these resistant infections [24]. This is a slow and silent crisis, comparable to climate change. It is sometimes difficult to grasp the urgency of the situation, as the effects are not always immediately visible, but they are very real and getting worse.

**Figure 5:**
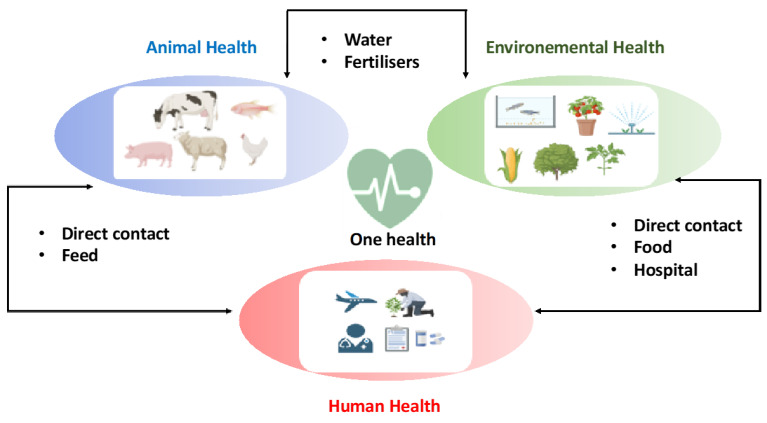
One Health concept: a single health for humans, animals and environment.

All research agrees on the dramatic consequences of this problem if no solution is found. As mentioned in the introduction, by 2050, antibiotic resistance could cause up to 10 million deaths per year [8]. This is higher than the current total number of deaths from cancer and infectious diseases. This is a silent pandemic and a real systemic, global, and interconnected threat. Antibiotics are widely used in intensive livestock farming, not only to treat infections, but also to prevent them and accelerate animal growth. These practices contribute to the spread of resistance in the environment, including in marine environments. It is therefore a global threat that affects our health, our food, and our ecosystems. It is precisely for this reason that the World Health Organization now refers to the “One Health” approach: one health for humans, animals and environment [25] (Figure 5).

The One Health concept emerged in the 2000s in response to the sharp increase in infectious diseases. It reflects the desire to view health as three interdependent systems: animal health, human health, and environmental health. This concept naturally extends to the local, national, and global levels. Issues related to antibiotic resistance fit perfectly into this vision. As mentioned above, intensive agriculture and groundwater pollution from human and animal waste contribute to resistance. It has been shown that residues of unmetabolized drugs or primary and secondary metabolites end up in wastewater and are not all destroyed by wastewater treatment, thus exposing the entire population to excessive contact [26,27]. All these phenomena are therefore linked and must be addressed as a whole. This obviously concerns not only antibiotics, but also many other hormone based pharmaceuticals, pesticides and insecticides that contaminate all terrestrial and marine resources. It is therefore necessary to adopt a multifactorial approach to these problems and propose interdisciplinary research.

### 2.1. Antimicrobial Peptides (AMPs)

**Figure 6:**
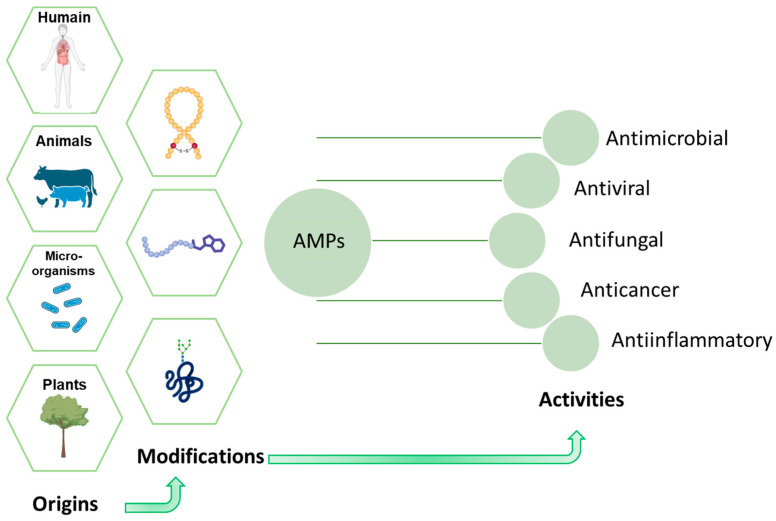
AMPs Properties.

Faced with this situation and with regard to the subject at hand, continuing as before is no longer an option. It is becoming urgent to rethink our weapons against bacterial infections. Among the most promising solutions today are antimicrobial peptides (AMPs) which represent a new generation of natural molecules with innovative mechanisms of action capable of circumventing the resistance developed by pathogenic bacteria [28]. These peptides, typically comprising 10–50 amino acid residues and carrying net positive charges, present a wide structural diversity [29]. They are produced naturally by all living organisms and they are part of the immune system’s first line of defense a kind of natural shield against infection in the immune system [30,31,32]. As a result of their varied origins, they possess extraordinary natural chemical diversity, endowing them considerable potential and a broad spectrum of biological activities [33,34]. In addition to their antibacterial properties, some AMPs are active against various pathogens. They have antiviral, antifungal, anti-inflammatory and even anticancer properties [35] (Figure 6). They regulate the immune system by increasing the expression of pro-inflammatory cytokines [32]. They are often referred to as a true therapeutic Swiss Army knife.

A major advantage of these AMPs lies in their multiple mechanisms of action, whose biological targets differ from those of conventional antibiotics [36]. Unlike conventional antibiotics, which generally target specific cellular functions such as DNA synthesis, the main action of AMPs occurs at the level of the bacterial cell membrane [30,37]. They insert themselves into the membrane, forming pores [32], which leads to permeability problems and the leakage of metabolites. All of this leads to the death of bacterial cells. They interact with the overall physicochemical properties of membranes, such as charge and hydrophobicity, limiting the development of specific resistance mechanisms [38]. This characteristic gives AMPs great potential against multidrug-resistant pathogens, especially since no significant increase in bacterial resistance to these peptides has been reported to date [39].

To date, few AMPs have passed the major regulatory hurdles. The most advanced example appears to be magainin, an amphiphilic α-helical membranolytic peptide composed of 23 residues derived from the skin of *Xenopus laevis* [40]. This peptide, developed by Magainin Pharmaceutical Inc. in the 1990s, reached phase III clinical trials for the treatment of foot ulcers in diabetic patients. However, it was rejected by the FDA because its efficacy was deemed insufficient compared to current standards [41,42].

Currently, other candidates derived from other organisms with varied structures are still in the early stages of development. These include brevinins, amphiphilic α-helical from the skin of *Limnonectes fujianensis* [43]; plectasin, a-β-stabilized fungal defensin isolated from the fungus *Pseudoplectania nigrella* [44] and protegrin 1, a-β hairpin cysteine rich and derived from pigs [45]. All have a broad spectrum of activity and show encouraging results in both in vitro and phase I/II clinical trials. However, as always, they face persistent challenges such as toxicity, stability, formulation issues, and, of course, manufacturing costs. This highlights that, beyond their undeniable biological properties, the industrial feasibility and economic viability of their production remain a major obstacle. The central question is, therefore, given the diversity of structures and origins, combined with these limitations: what production approach could overcome these obstacles and offer a realistic path towards clinical progress?

#### 2.1.1. Chemical Synthesis Approach

**Figure 7:**
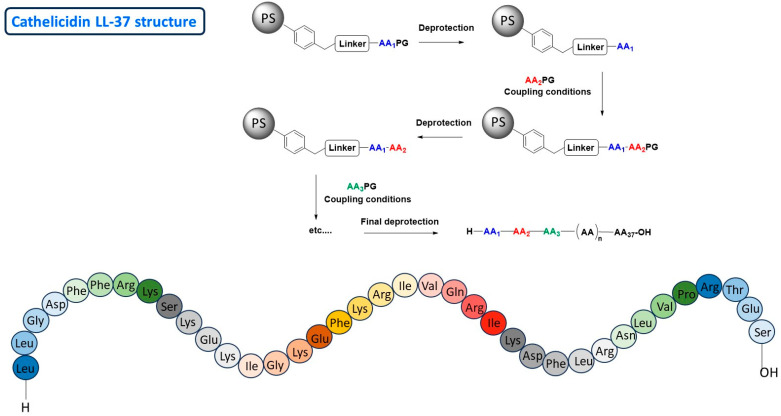
Cathelicidin LL-37 structure.

Peptides are obtained by chemical synthesis in a solid phase. Since the 1960s, this supported peptide synthesis was made possible by the pioneering work of Bruce Merriefield [46], this method has seen significant progress and has continued to improve ever since. Indeed, automation (synthesis or purification), the discovery of more environmentally friendly solvents, and the commercial availability of natural and non-natural amino acids, both protected and unprotected, offer significant advances in optimizing the activity, stability, and immunogenicity of peptides. For instance, the peptide LL-37 is a human cathelicidin peptide human [32]. It exhibits antimicrobial activity and also immune modulation and antibiofilm activity [47]. As mentioned above, LL-37 also modulates pro-inflammatory and anti-inflammatory immune responses in inflammatory diseases such as rosacea and psoriasis [48]. APMs generally have broad-spectrum antimicrobial activity, and this is also the case for LL-37 [49]. They can be effective against other organisms such as enveloped and non-enveloped viruses, yeasts, fungi, molds, etc. [36,50,51]. LL-37 is composed of 37 amino acids, and its chemical synthesis requires more than 40 steps. This chemical synthesis is usually carried out on a polymer support [52] and the number of steps corresponds to the number of amino acids to be incorporated into the chain, plus the steps required to attach and cleave the compound from the solid support (polystyrene: PS) and the various deprotection steps to remove the protecting groups (Figure 7). Usually Fmoc: 9-fluorenylmethoxycarbonyl or tert-butyloxycarbonyl is used as protected amino acids reagents. This synthesis was also carried out under microwave irradiation using ChemMatrix resin and optimizing conventional peptide coupling reagents as Hexafluorophosphate Azabenzotriazole Tetramethyl Uronium (HATU), Hexafluorophosphate Benzotriazole Tetramethyl Uronium (HBTU) or *N*,*N*′-Diisopropylcarbodiimide (DIC) [53]. The yield is lower when the number of amino acids involved and/or the structural complexity increases. Although the synthesis process has been automated, production costs remain high, mainly due to the high cost of the reagents and solvents required for large-scale production. The same applies to the purification steps of the final product after cleavage of the solid support. For complex AMPs, the economic equation for their production therefore becomes difficult to maintain through chemical synthesis. These limitations justify the search for alternative approaches capable of absorbing structural complexity without increasing costs.

#### 2.1.2. Bioproduction Approach

The recombinant production of these compounds represents a promising biological alternative. It relies on the use of living organisms, mammals, bacteria, yeasts, or plant cells, to efficiently express recombinant biomolecules, including peptides, and is particularly suited to large-scale production [54]. This is referred to as a “cell factory” capable of producing recombinant AMPs that are often difficult to synthesize chemically, while reducing the costs and environmental impact of their production [55]. This approach circumvents certain limitations of organic synthesis for the development of long and/or complex peptides and facilitates the transition to industrial scale. For example, the peptide LL-37, or more precisely its recombinant form GLL-37, has recently been produced using *E. coli*. However, although its production was efficient and performed in large quantities, GLL-37 unfortunately does not exhibit the activities of LL-37 [56]. The choice of host is therefore particularly strategic. While bacterial production is particularly well suited to certain peptides, this is not the case for all of them. Peptides that are more complex in terms of length or the presence of various disulfide bridges or glycosylation sites require the use of other host systems such as *Pichia pastoris* yeast for molecules such as plectasin [57] and *Saccharomyces cerevisiae* yeast for the peptide ETD-151 (fungal defensin) [58]. Mammalian cell lines (CHO, HEK293) are the gold standard for the production of clinical-grade biopharmaceuticals, and are the most commonly used. Nevertheless, they have high operating costs and an unfavorable environmental footprint due to their consumption of energy, water, and complex culture media [55]. However, this method remains expensive. The production of antibodies by these cells can cost several thousand euros per gram. For example, the cost is around 70,000$ per year for treatment with the cancer antibody trastuzumab (Herceptin^®^) produced in recombinant Chinese hamster ovary cells [59,60]. It would therefore be advantageous to have more sustainable, economical, and scalable production platforms to meet the objectives of the “One Health” program. What if the solution came not from chemistry or mammalian cells, but from the ocean, thanks to microorganisms capable of transforming sunlight into medicines? This is exactly what microalgae offer. In recent years, researchers have been exploring this possibility given the great diversity of these microalgae and their potential [61].

### 2.2. Microalgae

Aquatic ecosystems host a wide variety of living organisms classified as phytoplankton, which includes microalgae and cyanobacteria, two photosynthetic organisms. These microorganisms are very interesting because they harness sunlight and use atmospheric carbon dioxide to release oxygen into the atmosphere [62]. Owing to their highly efficient photosynthesis, microalgae contribute to nearly 40% of marine primary production and generating approximatively one-fifth of the oxygen we breathe [63]. Their extraordinary diversity is reflected in the more than one million species cataloged to date [64] as well as in their ability to colonize a wide range of environments, from marine and freshwater habitats to snowfields and even in hot springs [65]. This ecological versatility stems from metabolic flexibility, inherited from their unique evolutionary history shaped by endosymbiosis theory [66]. This explains how we distinguish several major taxonomic groups, including green microalgae, red microalga and diatoms [67]. Each has its own characteristics and specific ecological roles [68].

#### 2.2.1. Microalgae as Sustainable Biotechnology Platforms: Cultivation, Safety and Biotechnological Potential

Recent technological advances have significantly accelerated the development of microalgae cultivation. The use of photobioreactors enables controlled production can now be conducted in efficient and large-scale manner [69]. Microalgae are particularly attractive as a production platform due to their high growth rates (average doubling time of 1–2 days), high biomass yield (estimated around 0.9 to 1 euro/kg of dry biomass) [70]. These advantages stem from their ability to grow autotrophically using sunlight and atmospheric CO_2_ with minimal nutrient requirements. In contrast, conventional cell systems such as mammalian cells do not generate usable biomass and are dedicated solely to recombinant protein production. Their operational costs are significantly higher, often ranging from 50€ to 2000€ per gram of purified protein depending on the level of refinement and compliance with Good Manufacturing Practices (GMP) standards [71]. This difference highlights the economic and environmental advantages of microalgae, both for their integration into circular bioeconomy models and for the development of environmentally friendly bio-manufacturing strategies [63].

Beyond their advantages in terms of cultivation, they appear to be an effective and safe platform for the production of heterologous proteins due to the absence of common human pathogens and their classification as “Generally Recognized as Safe” (GRAS) [72,73,74,75,76,77]. In addition, their extensive phylogenetic diversity offers many opportunities for the development of new molecules of biotechnological and pharmaceutical interest. This potential can be further enhanced through genetic engineering [77].

#### 2.2.2. Microalgae Platforms for AMP Expression: Comparative Overview

Recent research in bioproduction has highlighted the use of microalgae to generate recombinant molecules including AMPs [78]. Advances in genetic engineering tools and the availability of low-cost culture systems have further accelerated progress in this field [77,79]. They are therefore attracting growing interest in the development of transgenic strains capable of producing recombinant bioactive compounds with enhanced therapeutic efficacy, while maintaining high standards of safety and reliability [61,80]. Nevertheless, while microalgae offer unique advantages as recombinant production platforms, their application to antimicrobial peptide synthesis remains in its beginning [81].

**Figure 8:**
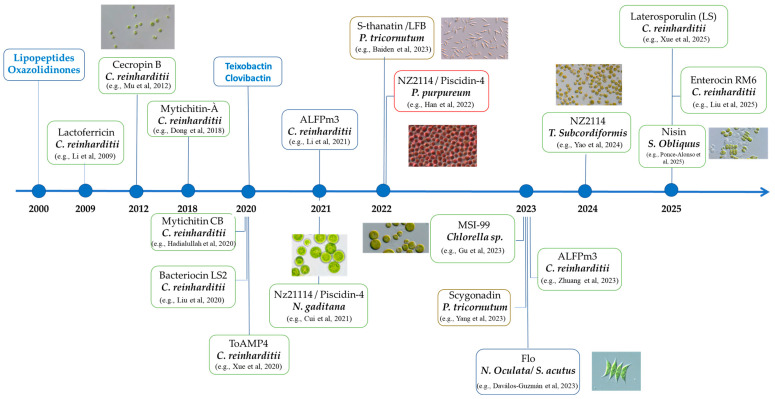
Examples of the Evolution of Research on AMPs Production in Microalgae [83,84,85,86,87,88,89,90,91,92,93,97,98,99,100,101,113].

A closer look at recent examples and a comparison with other established cell-based systems is essential to fully assess both their potential and challenges that must be addressed to optimize their performance. Several microalgae species have been successfully engineered to produce AMPs, among them *Chlamydomonas reinhardtii*, a green microalga, stands out as a pioneer host for recombinant protein expression. Its appeal lies in its well characterized genetics, the availability of both nuclear and chloroplast transformation systems and its ease of cultivation. Initially developed as a model for molecular and cellular research, it has since been used for AMP production [82,83]. It has also been used to express peptides such as Cecropin B, Enterocin RM6 and ALFPm3 with confirmed antimicrobial activity against Gram-positive and Gram-negative bacteria [84,85,86]. Reported expression levels typically range from 1 to 2 mg/L in culture depending on the construct and the targeted compartment [83]. Beyond *C. reinhardtii*, other microalgae species have also been explored for the targeted AMPs biosynthesis (Figure 8) [87,88,89,90,91,92,93].

For example, the red microalgae *Porphyridium purpureum*, was used to biosynthesize the peptide NZ2114, an analog of the fungal defensin plectasin using nuclear transformation and secretion signals to facilitate peptide recovery. Although the reported yield is lower (≈1.2 mg/L) the extracellular recovery simplifies downstream processing [94]. In comparison, the same peptide produced in *Pichia pastoris* yielded up to 1 g/L, highlighting the gap in productivity and the advantage of extracellular secretion in microalgae [95]. NZ2114 was also expressed in mammalian systems such as CHO cells, where the focus is on achieving pharmaceutical-grade purity and human compatible post translational modifications. Despite lower yields than in yeast or bacterial systems, the regulatory compliance and therapeutic relevance justify the use of such platforms for clinical applications [96].

Another promising model is the diatom *Phaeodactylum tricornutum*, whose biotechnological potential has expanded rapidly following the availability of its genome sequence and advance in genomic annotation, codon optimization and efficient genetic transformation techniques. Initially explored for the production of recombinant antibodies, *P. tricornutum* has more recently been used to produce two recombinant AMPs, the bLFcin (a bovin Lactoferricin), S-thanatin (an insect *Podisus maculiventris*) and a secreted AMP (from *Sylla serrata*) with expression confirmed at the protein level and bioactivity demonstrated in antimicrobial assays [97,98].

Other species like *Nannochloropsis oculata* and *Chlorella sp.* have also been explored for AMP expression, either through chloroplast or nuclear transformation. Recombinant production of some peptides was demonstrated in these hosts although yields remain modest and vary depending on the transformation strategy and cultivation conditions [99,100]. More recently, *Scenedesmus obliquus* has been used to produce the AMP Nisin, a well characterized member of the lantibiotics AMPs, using nuclear transformation strategies. Her again, although yields remain modest, the successful expression and bioactivity of Nisin in this species further expands the repertoire of microalgae hosts for AMP production [101]. These examples illustrate the diversity of microalgae hosts available for AMP production, each offering advantages in terms of transformation efficiency, secretion capacity and cultivation conditions The choices of species and expression system depend on the target peptide, desired yield and downstream application.

As already mentioned, many of these AMPs have also been produced in conventional systems such as *E. Coli*, *Pichia pastoris* and mammalian cells, often with higher yields but requiring more complex purification and posing biosafety concerns. Comparing expression across platforms highlights the trade-offs between productivity, safety and sustainability. However, beyond yield and biosafety, post translational modifications and particularly glycosylation must be considered and measured when microalgae were evaluated as production hosts. This modification is essential for the biological activity and the stability of most therapeutic proteins. While mammalian cells naturally perform human compatible glycosylation, microalgae have divergent glycosylation pathways that can unfortunately limit their pharmaceutical applications. Some studies have explored this phenomenon in various species, including *C. reinhardtii*, *P. tricornutum*, *C. vulgaris*, *Botryococcus braunii*, *Dunaliella salina* and *Porphyridium sp.*, revealing a wide range of *N*-glycan structures. These are often oligomannosidic, methylated, or contain unusual residues such as arabinose and xylose [102,103,104,105,106,107].

Even within the same species, C. Toustou et al. showed in 2024 [80] that glycosylation profiles could vary between ecotypes, highlighting the influence of genetic and epigenetic background. These non-human glycan structures can affect protein folding as well as serum half-life and immunogenicity, particularly in the pharmaceutical context. To overcome these limitations, glycoengineering strategies have been developed, notably in *C. reinhardtii*, to introduce human glycosyltransferases and partially humanize glycan profiles [102]. However, greater efforts are needed to map and modify glycosylation pathways in various microalgal hosts. It is therefore essential to understand the underlying biosynthetic pathways. Targeted glycoengineering is necessary to develop humanized strains. To advance this field, it would be necessary to deepen the understanding of species-specific biosynthetic pathways and develop standardized tools for glycan analysis and pathway manipulation. This remains a critical area of research to position microalgae as viable platforms in pharmaceutical biotechnology.

#### 2.2.3. Application of Microalgae Derived AMPs Towards Multi-Sector Exploitation

Antimicrobial peptides derived from microalgae are a promising biotechnological resource with applications in therapeutic, cosmetic, and environmental biotechnology fields. However, for their pharmaceutical exploitation to be viable, production yields must be significantly improved. This requires the use of powerful promoters, suitable expression vectors with a better understanding of transcriptional and post-translational regulatory mechanisms and optimized signal peptides to ensure protein secretion in the culture medium. This avoids complex purification steps and yield losses [108,109,110]. Beyond traditional therapeutic applications, AMP-enriched microalgae could also offer significant potential in the field of environmental biotechnology. One particular example is the biological control of biofilms and the natural disinfection of aquatic systems [111]. In the cosmetics industry, algae extracts containing antimicrobial peptides can serve as natural preservatives, meeting the demand for clean, bio-based formulations [112]. Although microalgae protein yields are lower, non-therapeutic sectors offer the possibility of using AMP-producing microalgae without purification. Economic viability is enhanced when the biomass itself serves as a delivery vehicle, particularly in aquaculture, food, or nutraceutical applications [78,113]. This positions microalgae as a sustainable alternative to recombinant AMP production in contexts where ultra-purification is not necessary [114]. These various potential applications highlight the strategic value of AMPs derived from microalgae. Their optimal exploitation depends on the ability to overcome current limitations related to biosynthetic efficiency, purification processes, and precise regulation of expression systems. Addressing these challenges will be essential to unlocking the potential of microalgae in next-generation biopharmaceutical and bio industrial platforms.

## 3. Conclusions

In conclusion, antimicrobial peptides (AMPs) appear to be key products in the fight against antibiotic resistance. The choice of production methods, whether chemical, biological, or derived from innovative systems, is a decisive factor in meeting growing demand. We believe that microalgae represent a very promising alternative to conventional cell bioreactors, particularly mammalian cell lines, with often significantly lower production costs. However, further studies are needed to improve production yields and process design. Combined with advances in genetics and molecular biology, this could lead to stable, high-performance strains that could become the “factory cells” of tomorrow. Focusing on these areas of development could transform this promise into a sustainable industrial solution that can be applied on a large scale.

## Data Availability

No new data were created or analyzed in this study. Data sharing is not applicable to this article.
